# Genetic diversity of respiratory syncytial virus in children with community-acquired pneumonia in Guangzhou: an epidemiological update

**DOI:** 10.1038/s41390-025-04214-7

**Published:** 2025-07-01

**Authors:** Guixing Zheng, Changwen Zhan, Hainei Pan, Yingfang Lu, Haiqi Zhang, Xueying Xu, Xinyuan Lin, Sheng Qin

**Affiliations:** 1 Department of Blood Transfusion, The First Affiliated Hospital of Guangzhou Medical University, Guangzhou, China; 2 Department of Clinical Laboratory, Guangzhou Hospital of Integrated Traditional and Western Medicine, Guangzhou, China; 3 Department of Medical Laboratory Science, Guangzhou University of Chinese Medicine, Guangzhou, China; 4 Department of Clinical Laboratory, Guangdong Provincial Hospital of Chinese Medicine, Guangzhou, China

## Abstract

**Background:**

Community-acquired pneumonia (CAP) is a common infectious disease with a mortality rate of 2–14%. Respiratory syncytial virus (RSV) frequently causes CAP in children. This study aimed to investigate RSV genetic diversity in children under 15 years of age with CAP, evaluate nucleotide substitution rates, and examine associations with clinical characteristics and outcomes.

**Methods:**

A retrospective observational study was conducted using nasal swab samples collected from children under 15 years of age diagnosed with CAP. RSV identification involved cell culture and immunofluorescence microscopy, followed by molecular analyses using qPCR, nested PCR, and Sanger sequencing. Bioinformatics tools were applied to assess phylogenetics, evolutionary trends, genetic distance, and nucleotide substitution rates. Statistical analysis of clinical and laboratory data was performed to identify correlations between RSV features and patient characteristics.

**Results:**

Among 346 CAP cases, RSV was detected in 26.88%, with the highest prevalence in children aged 1–2 years. Subtype A exhibited higher nucleotide substitution rates than subtype B. Shortness of breath and high fever were significantly associated with increased nucleotide diversity. RSV infections peaked in winter, particularly in 2018 and 2019. Elevated substitution rates were linked to longer hospital stays, greater risk of complications such as secondary bacterial infections, and increased RSV-related morbidity.

**Conclusion:**

RSV higher nucleotide substitution rates, especially in children under 3 years of age, were associated with more severe clinical outcomes and prolonged hospitalization. Phylogenetic analysis indicated distinct evolutionary patterns, with subtype B exhibiting a more rapid evolutionary rate than subtype A.

**Impact:**

- This study highlights the high prevalence of respiratory viruses, particularly respiratory syncytial virus (RSV), in children with community-acquired pneumonia in Guangzhou. It provides detailed molecular epidemiological data on RSV, including genetic variation patterns and evolutionary rates. The findings contribute regional data essential for epidemiological research and public health planning in similar geographic areas. These results support clinical decision-making and inform public health strategies, particularly those focused on RSV vaccination and infection control.

## Introduction

Community-acquired pneumonia (CAP) is a common respiratory infection acquired outside hospitals or healthcare settings. It is characterized by lung inflammation. Common symptoms include fever, cough, difficulty breathing, and chest pain. CAP remains one of the leading causes of hospitalization among children in high-income countries^1^ and a major cause of death among children in low- and lower-middle-income countries.^2^

Viruses are the most frequent cause of CAP in children, with respiratory syncytial virus (RSV) accounting for most cases.^1,2^ RSV is most prevalent among young children, contributing to ~50% of CAP cases in those under 5 years of age. Among these, 88% occur in children under 2 years. RSV leads to nearly 3.4 million hospitalizations and ~70,000 deaths in children each year.^3^ Infants account for the largest number of RSV cases requiring medical attention.^4–7^

RSV strains are classified into RSV-A and RSV-B subtypes based on antigenic and genetic differences.^8^ These subtypes typically cocirculate in human populations, and both can cause severe disease. Studies have reported considerable variation in the clinical severity associated with each subtype.^8^ A prior study on Chinese children (≤14 y of age) with respiratory infections between September 2017 and December 2021 identified 93 RSV sequences, including 56 (60.2%) classified as RSV-A and 37 (39.8%) as RSV-B. No coinfections involving RSV-A and RSV-B were observed in nasopharyngeal (NP) swab samples. Phylogenetic analysis revealed that RSV-A and RSV-B strains belonged to genotypes ON1 and BA9, respectively, indicating the predominance of these genotypes in Guangzhou, China. After 2019, notable changes in respiratory pathogen profiles were reported following the emergence of COVID-19. These changes were likely influenced by prevention practices such as face masks, hand hygiene, and social distancing.^9–12^ Evaluating CAP epidemiology before and during the pandemic is therefore essential.

Understanding the longitudinal epidemiology of CAP is vital for monitoring viral evolution and informing public health interventions. A previous study in Guangzhou investigated CAP trends in children from 2010 to 2019, identifying seasonal patterns and dominant pathogens during that time.^13^ Building upon those findings, the current study explores the clinical features, molecular epidemiology, and genetic diversity of RSV in CAP cases from 2018 to 2020. This investigation offers updated insights into the changing landscape of pediatric respiratory infections. The study also examined pathogen distribution, seasonal variation, and epidemic patterns, focusing on genetic variability, evolutionary rates, and selective pressures of RSV subtypes. Finally, associations between RSV subtypes and clinical or hematological features, along with disease severity, were analyzed.

## Materials and methods

This retrospective study was conducted on 346 patients who presented with acute respiratory symptoms such as fever, cough, shortness of breath, or laboratory-confirmed evidence of respiratory infection. Patients were recruited at Guangdong Provincial Hospital of Traditional Chinese Medicine (Approval number: ZE2020-3034-01) between January 2018 and December 2020.

### Study population

Patients were admitted to or visited Guangdong Provincial Hospital of Traditional Chinese Medicine between January 2018 and December 2020. Patients were included if they met the following diagnostic criteria: 1) Symptoms of acute infection such as fever, abnormal white blood cell counts, chills, or altered body temperature. 2) Respiratory signs included cough, sputum production, shortness of breath, abnormal breath sounds, or chest pain. 3) Chest X-ray findings indicating inflammatory changes such as patchy or consolidative opacities. The exclusion criteria included incomplete medical records and conditions unrelated to CAP, such as pulmonary tuberculosis, lung tumors, or pulmonary embolism.

The control population consisted of nasal swabs from patients who were hospitalized or visited the outpatient clinic due to acute infection without respiratory symptoms but underwent respiratory virus culture and identification testing. It is important to note that these tests were not performed for all hospitalized patients, particularly those without respiratory symptoms. Therefore, the number of non-CAP control samples was limited. The control population was evaluated to compare clinical and laboratory markers of CAP with those of other acute illnesses.

### Specimen collection

Nasal swabs were collected by inserting a sterile swab into the nasopharynx, rotating it for 5–10 s, and sealing the specimen for transport. Samples were either tested immediately or stored at 4 °C and analyzed within 24 h. After shaking and centrifugation, the supernatant was divided for cell inoculation and molecular testing.

### Laboratory analyses

#### Virus cultivation

The specimens were inoculated into three cell lines: Madin-Darby canine kidney (MDCK), Rhesus monkey kidney epithelial (LLC-MK2), and human epithelial (HEp-2). Cell cultures were prepared by seeding 1 × 10^5^ cells/mL into 96-well plates. Plates were incubated at 37 °C with 5% CO_2_ until a confluent monolayer formed. After discarding the culture medium, cells were rinsed twice with phosphate-buffered saline (PBS) and inoculated with 100 μL of the processed sample. MDCK and LLC-MK2 cells were supplemented with 4% TPCK (tosyl phenylalanyl chloromethyl ketone) medium. HEp-2 cells received 4% DMEM (Dulbecco’s Modified Eagle’s Medium). Cultures were incubated at 37 °C with 5% CO_2_ for 5 d. They were monitored daily for cytopathic effects (CPE) and analyzed by immunofluorescence on the fifth day.

#### CPE interpretation

**Fig. 1:**
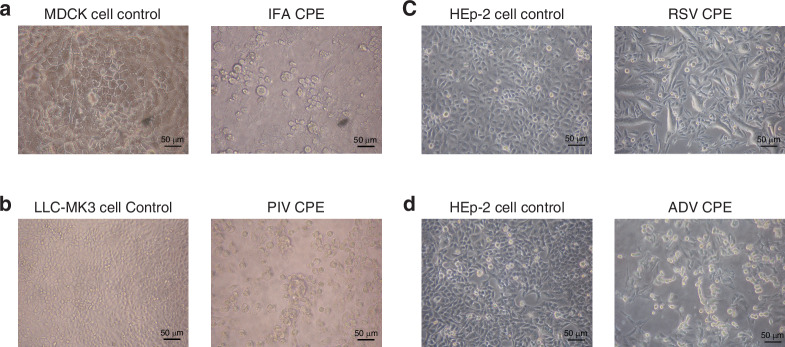
Cytopathic effects were observed in different cell lines. **a** Influenza A virus (IFA), **b** Parainfluenza virus (PIV), **c** Adenovirus (ADV), and **d** Respiratory syncytial virus (RSV).

MDCK cells were used to observe CPE induced by influenza A virus (IFA) and influenza B virus (IFB). Infected cells exhibited rounded morphology, increased refractivity, and a fragmented CPE pattern (Fig. 1a). LLC-MK2 cells were used to observe CPE caused by the parainfluenza virus (PIV). Infected cells displayed rounded morphology, increased refractivity, and a fragmented CPE pattern. Specific PIV types could not be distinguished by morphology (Fig. 1b). HEp-2 cells were used to observe CPE induced by RSV and adenovirus (ADV). These CPE types were morphologically distinct. RSV infection caused syncytial giant cell fusion. ADV infection produced bead-like or grape-like lesions (Fig. 1c, d).

#### Immunofluorescence

Cultured specimens were converted into cell suspensions within their respective wells. Samples from the three cell types were transferred onto a multi-well glass slide, with 2 drops per well, each measuring 22 μL. The slide was dried in an oven at 42 °C and then processed using immunofluorescence staining according to the reagent instructions. Stained slides were examined using an upright fluorescence microscope.

Under 200× magnification, the presence of two or more green fluorescent cells was considered positive, while fewer than two fluorescent cells indicated a negative result. Negative cells were counterstained with Evans Blue and appeared red. Virus-specific fluorescence patterns were observed as follows. IFA and IFB produced cytoplasmic, nuclear, or mixed fluorescence staining. Cytoplasmic staining exhibited granular fluorescence with large inclusions, whereas nuclear staining appeared bright and uniformly fluorescent. RSV generated cytoplasmic fluorescence with granular characteristics and small inclusions near syncytial junctions. ADV showed granular cytoplasmic fluorescence, bright nuclear fluorescence, or both. PIV exhibited cytoplasmic fluorescence with granular features and irregular inclusions. Types 2 and 3 occasionally induced syncytium formation.

### Bioinformatics

RSV nucleic acid extraction, quantitative PCR (qPCR), nested PCR, and Sanger sequencing were performed according to the method described by Umar et al.^14^. Viral sequences were subjected to phylogenetic, evolutionary, and genetic distance analyses using tools such as TempEst, MEGA11, and Shell. Substitution rates were determined using the second variable region of the RSV G protein.

#### Phylogenetics

TempEst was used to perform a time-to-most-recent-common-ancestor (TMRCA) analysis. Selection pressure was assessed using the single likelihood ancestor counting (SLAC) method and the mixed effects model for episodic diversifying selection (MEME), which identified positively and negatively selected sites. Pairwise genetic distances were calculated using the Kimura 2 model. Average and standard deviation values for nucleotide and amino acid distances were computed for RSV-A and RSV-B samples, based on both full-length sequences and the second variable region. RSV-A and RSV-B sequences were trimmed at the second variable region of the G protein to obtain the HVD2 sequence. ON1 and BA9 sequences were used as references. Shell was used to estimate base substitution rates, and bar graphs were generated using the R software package ggplot2.

### Statistical analysis

Quantitative data are reported as mean ± standard deviation (SD), and frequencies with percentages were used for categorical variables. Variance tests were conducted for quantitative variables such as patient age and laboratory test results. Quantitative variables were analyzed using analysis of variance (ANOVA), while categorical variables were analyzed using chi-square tests. A *p*-value < 0.05 was considered statistically significant. Statistical analysis was performed using SPSS version 22.0 software.

## Results

### Clinical and laboratory characteristics of children with CAP

Among children diagnosed with CAP, 60.1% (*n* = 208) were male. The average age was 3.68 years, and the largest proportion (30.06%) were aged 1–2 years. No newborns were included. Most children (87.78%) exhibited moderate to high fever (>38 °C), and several had temperatures ranging from 39.1 to 41 °C. Table 1 summarizes the general characteristics of children with CAP.

**Table 1:** summarizes the general characteristics of the children with CAP.

**Gender**	
Male	208 (60.1)
Female	138 (39.9)
Age (mean ± SD)	3.68 ± 3.22
Age Groups (*n*, %)	
Infant (1–12 months)	55 (15.90)
Toddler (1–2 years)	104 (30.06)
Preschooler (3–5 years)	101 (29.19)
School-aged child (6–12 years)	77 (22.25)
Adolescent (13–17 years)	9 (2.60)
Mean Highest Temperature (°C) (mean ± SD) *n* = 319	
Highest Temperature groups (°C) (*n*, %)	39.21 ± 0.93
<37.3	15 (4.70)
37.3–38 (Low-grade fever)	24 (7.52)
38.1–39 (Moderate fever)	94 (29.47)
39.1–41 (High fever)	181 (56.74)
>41 (Very high fever)	5 (1.57)
Mean length of stay (*n* = 342) (days)	6.43 ± 3.36
Frequency of children with normal respiratory rate (*n*, %)	239 (69.9)
Infant (1–12 months)	35 (66.00)
Toddler (1–2 years)	76 (73.10)
Preschooler (3–5 years)	68 (68.70)
School-aged child (6–12 years)	60 (77.90)
Adolescent (13–17 years)	0 (0.00)

The most frequently reported symptoms among children with CAP were cough (94.51%), sputum production (78.90%), rhinorrhea (51.45%), and nasal congestion (49.71%). Other symptoms such as wheezing, dyspnea, and poor appetite were also significantly more common in CAP cases compared to children with other acute illnesses. In addition, children with CAP had significantly lower white blood cell counts (*P* < 0.01), neutrophil percentages (*P* = 0.01), lymphocyte percentages (*P* = 0.048), hematocrit (*P* < 0.001), and hemoglobin levels (*P* < 0.001). In contrast, monocyte percentages were significantly higher (*P* < 0.001). Serum amyloid A (SAA), C-reactive protein (CRP), procalcitonin (PCT), and ketones were elevated in both groups. However, no statistically significant differences were observed. Table 2 outlines the clinical features and laboratory findings of children with and without CAP.

**Table 2:** Analysis of clinical symptoms and laboratory indicators results in all children and RSV-positive CAP children.

	CAP children (*n* = 346)	Children without CAP (*n* = 104)	Odds Ratio (OR)/*P* value	RSV A Group (*n* = 56)	RSV B Group (*n* = 37)	*P* value
Age groups						
Infant (1–12 months)	–	–	–	19	10	0.214
Toddler (1–2 years)	–	–	–	26	15	
Preschooler (3–5 years)	–	–	–	10	11	
School-aged child (6–12 years)	–	–	–	1	0	
Clinical symptoms						
Fever	–	–	–	53/3	32/5	0.320
Respiratory rate (normal/abnormal)	–	–	–	39/17	27/10	0.729
Chills	28 (8.09)	2	4.491 (1.051–19.178) /0.027*	0/56	3/34	0.060
Shivering	7 (2.02)	0	0.980 (0.965–0.995) /0.144	2/54	0/37	0.516
Dizziness	3 (0.87)	7	0.121 (0.031–0.478) /<0.001*	0/56	0/37	–
Headache	8 (2.31)	4	0.592 (0.175–2.006) /0.395	0/56	0/37	–
Fatigue	7 (2.02)	2	1.053 (0.215–5.149) /0.949	0/56	1/36	0.398
Muscle soreness	4 (1.16)	0	0.988 (0.977–1.000) /0.271	2/54	0/37	0.516
Nasal congestion	172 (49.71)	17	5.059 (2.887–8.864) / < 0.001*	40/16	21/16	0.145
Runny nose	178 (51.45)	24	3.532 (2.137–5.838) / < 0.001*	41/15	25/12	0.557
Sneezing	7 (2.02)	1	2.127 (0.259–17.488) / 0.473	2/54	2/35	1.000
Dry mouth	8 (2.31)	2	1.207 (0.252–5.774) / 0.813	1/35	0/37	1.000
Sore throat	23 (6.65)	6	1.163 (0.461–2.937) / 0.749	2/54	1/36	1.000
Dry cough	10 (2.89)	2	1.518 (0.327–7.040) / 0.591	1/55	0/37	1.000
Cough	327 (94.51)	32	38.724 (20.782–72.154) /<0.001*	54/2	35/2	1.000
Sputum production	273 (78.90)	23	13.170 (7.750–22.381) /<0.001*	53/3	29/8	0.040*
Chest tightness	2 (0.58)	2	0.302 (0.042–2.173) / 0.208	1/55	0/37	1.000
Chest pain	4 (1.16)	0	0.988 (0.977–1.000) /0.271	0/56	0/37	–
Wheezing	40 (11.56)	0	0.884 (0.851–0.919) /<0.001*	9/47	5/32	0.736
Chest wheezing	7 (2.02)	0	0.980 (0.965–0.995) /0.144	0/56	3/34	0.060
Shortness of breath	110 (31.79)	5	9.229 (3.654–23.307) /<0.001*	32/24	14/23	0.068
Nausea	8 (2.31)	2	1.207 (0.252–5.774) /0.813	0/56	0/37	–
Vomiting	13 (3.76)	6	0.638 (0.236–1.722) /0.371	3/53	1/36	0.924
Poor appetite	140 (40.46)	27	1.938 (1.190–3.158) /0.007*	17/39	15/22	0.312
Abdominal pain	16 (4.62)	9	0.512 (0.219–1.195) /0.116	2/54	1/36	1.000
Diarrhea	10 (2.89)	2	1.518 (0.327–7.040) /0.591	4/52	0/37	0.148
Laboratory						
SAA(mg/L)	152.34 ± 172.65 (76)	80.13 ± 101.47 (10)	−/0.201	150.534 ± 221.750	33.353 ± 37.430	0.187
CRP(mg/L)	21.26 ± 28.41 (276)	20.84 ± 31.21 (61)	−/0.917	15.268 ± 18.703	12.913 ± 27.613	0.674
PCT(ng/mL)	1.03 ± 6.43 (321)	0.34 ± 0.54 (63)	−/0.402	0.545 ± 0.923	0.465 ± 1.188	0.721
Ketones(mmol/L)	0.79 ± 0.87 (127)	0.49 ± 0.37 (18)	−/0.156	1.385 ± 1.044	0.917 ± 0.900	0.244
WBC(×10^9^/L)	9.88 ± 5.10 (346)	11.70 ± 7.08 (103)	−/<0.01*	38/18	25/12	0.977
Neutrophils (%)	53.89 ± 19.12 (345)	59.41 ± 19.68 (103)	−/0.011*	32/24	20/16	0.881
Lymphocytes (%)	36.35 ± 18.12 (345)	32.35 ± 17.36 (103)	−/0.048*	44/12	29/7	0.819
Monocytes (%)	7.80 ± 3.01 (345)	6.14 ± 3.68 (100)	−/<0.001*	39/17	20/16	0.169
HCT (%)	36.64 ± 4.85 (336)	38.83 ± 5.74 (101)	−/<0.001*	39/17	31/4	0.037*
Hb(g/L)	121.06 ± 13.85 (341)	129.81 ± 21.07 (103)	−/<0.001*	24/32	24/13	0.038*
PLT (×10^9^/L)	339.24 ± 254.50 (343)	370.36 ± 134.11 (102)	−/0.236	18/38	25/11	<0.001*
nucleotide substitution rates (base substitutions/site/year)				2.6641 × 10^−3^	2.8067 × 10^−3^	–

*SAA* Serum Amyloid A, *CRP* C-Reactive Protein, *PCT* Procalcitonin, *HCT* Hematocrit.

^*^P value < 0.05.

### Pathogen Detection Rates and RSV Prevalence in CAP Cases

**Fig. 2:**
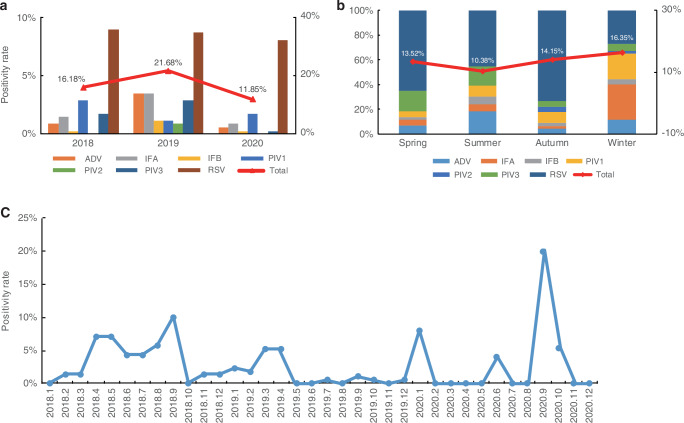
Epidemiology and seasonal trends of respiratory virus infections in children with CAP (2018–2020). **a** Distribution of respiratory virus infections in CAP cases from 2018 to 2020. **b** Seasonal trends of virus detection in CAP cases. **c** Monthly RSV positivity in CAP cases.

Among the 346 CAP cases collected, the total viral positivity rate was 50.86% (n = 176). Detection rates peaked in 2019 (21.68%) and declined in 2020 (11.85%), likely due to COVID-19 prevention strategies (Fig. 2a). RSV was the most prevalent virus (26.88%), followed by IFA (5.78%), parainfluenza virus type 1 (PIV1, 5.78%), PIV3 (4.91%), ADV (4.91%), IFB (1.73%), and PIV2 (0.87%) (Table 3). Further analysis indicated that RSV was most commonly detected in toddlers aged 1–2 years. The overall positivity rate for bacterial and fungal pathogens was 7.51%, with *Haemophilus influenza* (HFA) being the most frequently identified bacterial pathogen. Table 3 presents the viral and bacterial pathogen detection results.

**Table 3:** Analysis of pathogen testing results in CAP pediatric patients.

Pathogen	*n* (%)
Virus	176 (50.86)
RSV	93 (26.88)
Infant (1–12 months)	29 (31.18)
Toddler (1–2 years)	41 (44.09)
Preschooler (3–5 years)	21 (22.58)
School-aged child (6–12 years)	1 (1.08)
ADV	17 (4.91)
IFA	20 (5.78)
IFB	6 (1.73)
PIV1	20 (5.78)
PIV2	3 (0.87)
PIV3	17 (4.91)
Bacteria	26 (7.51)
Haemophilus influenzae	18 (5.20)
Staphylococcus aureus	4 (1.15)
Acinetobacter baumannii	3 (0.86)
Klebsiella pneumoniae	1 (0.27)
Pseudomonas aeruginosa	N

*N* Not detected.

### Seasonal trends of respiratory virus detection from 2018 to 2020

Data in Fig. 2b shows the seasonal distribution of respiratory viruses detected between January 2018 and December 2020. Respiratory viruses were most frequently detected in winter (16.35%), followed by autumn (14.15%), and were least common in summer (10.38%). RSV predominated in spring (65%), summer (45%), and autumn (73%). During winter, IFA had the highest detection rate (29%) with a positivity rate of 4.72%. RSV was the second most prevalent virus in winter, with a detection rate of 27% and a positivity rate of 4.40%. Other respiratory viruses, including IFB, PIV1, PIV2, PIV3, and ADV were detected consistently across the study period without distinct seasonal peaks. Further analysis of RSV trends showed that epidemics occurred over several months annually. RSV cases were observed from April to September in 2018, with peaks in March and April in 2019. In 2020, detection patterns became irregular due to the impact of COVID-19 (Fig. 2c).

### Molecular evolution and selection pressure of RSV subtypes

**Fig. 3:**
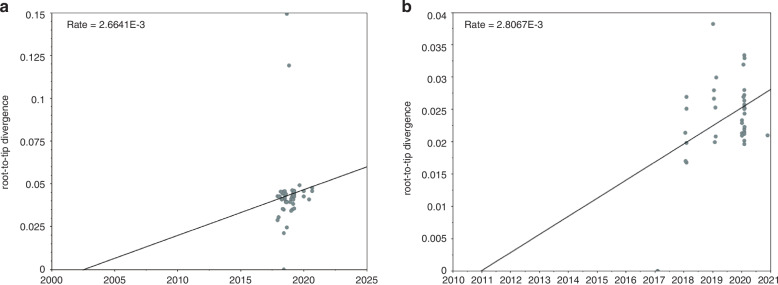
Evolutionary rate curves of RSV subtypes. **a** RSV-A and **b** RSV-B.

We examined the results of sequence alignment, phylogenetic analysis, amino acid sequence inference, and glycosylation site analysis of RSV using the methods of Umar et al.^14^. Phylogenetic analysis revealed that RSV-A and RSV-B exhibited comparable evolutionary rates. RSV-B evolved slightly faster (2.8067 × 10^−3^ substitutions/site/year) than RSV-A (2.6641 × 10^−3^ substitutions/site/year). Both subtypes underwent positive selection at multiple sites, suggesting ongoing viral adaptation (Fig. 3). Since the most recent common ancestor of subtype A predated that of subtype B, the evolutionary rate of subtype B was faster than that of subtype A. According to predictions from the SLAC model, neutral selection predominated in RSV subtypes A and B. Subtype A exhibited 67 positively and 61 negatively selected sites, while subtype B had 56 and 46, respectively.

Using the MEME, subtype A demonstrated a dominance of positively selected sites. Subtype B exhibited neutral selection as the main trend. Subtype A revealed 78 neutral selection sites and 128 positively selected sites. Subtype B had 145 neutral selection sites and 103 positively selected sites. Neither subtype showed any predicted negatively selected sites based on these analyses.

### Genetic distance, substitution rates, and clinical correlates of RSV subtypes

Genetic distance analysis was conducted on full-length nucleotide sequences and the nucleotide sequences of the second variable region of RSV subtypes A and B. For subtype A, the internal distance for full-length nucleotide sequences was 0.030 ± 0.027, while that for the second variable region was 0.029 ± 0.029. These values showed no significant difference. In subtype B, the distances were 0.024 ± 0.017 for full-length sequences and 0.022 ± 0.011 for the second variable region, with the full-length distance being greater.

Subtype B exhibited greater variability in full-length sequences. In contrast, the second variable region showed more variation in subtype A. For amino acid sequences, genetic distance was higher in full-length sequences than in the second variable region in both subtypes. Subtype A had distances of 0.071 ± 0.074 (full-length) and 0.054 ± 0.055 (second variable region). Subtype B had values of 0.146 ± 0.384 (full-length) and 0.045 ± 0.024 (second variable region).

**Fig. 4:**
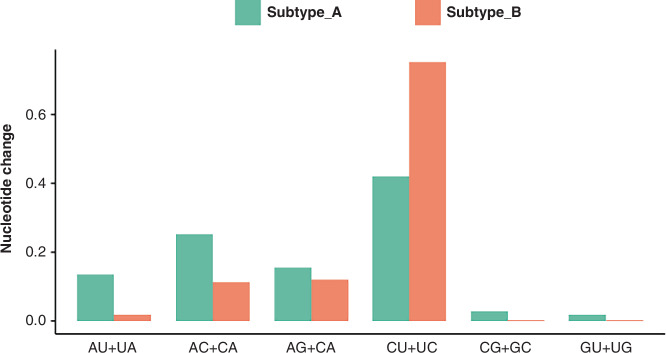
Nucleotide substitution rates in the second variable region of the RSV G protein gene (Subtype A).

Table 4 summarizes the prediction of positively and negatively selected sites for RSV strains based on SLAC and MEME methods. The substitution rate of the second variable region of the G protein gene was higher in subtype A. However, the transition rate was higher in subtype B. In both subtypes, the nucleotide transition rate exceeded the transversion rate. Subtype A had a transition-to-transversion ratio of 1.342, with CU + UC transitions predominating. Subtype B showed a higher ratio of 6.777, also dominated by CU + UC transitions. Notably, the transversion rates for CG + GC and GU + UG in subtype B strains were 0 (Fig. 4). Sputum production, hematocrit (HCT, %), hemoglobin (Hb, g/L), and platelet count (PLT) were significantly associated with RSV subtypes (all *P* < 0.05). RSV subtype diversity was more prominent in children aged 1–2 years, consistent with the higher RSV positivity rate in this age group (44.09%; Table 2).

**Table 4:** Prediction of common positively and negatively selected sites for RSV strains by both the SLAC and MEME methods.

Subtypes	Common positively selected pressure sites	Common negatively selected pressure sites
RSV-A	3, 5, 9, 11, 14, 16, 18, 19, 20, 21, 23, 27, 31, 34, 36, 37, 38, 40, 44, 50, 63, 73, 74, 76, 80, 84, 94, 95, 98, 100, 104, 107, 109, 110, 113, 117, 126, 132, 133, 136, 137, 138, 140, 141, 142, 143, 148, 150, 153, 155, 157, 159, 161, 169, 175, 176, 182, 183, 184, 188, 194, 195, 196, 197, 203, 204, 205	1, 2, 4, 7, 8, 13, 24, 28, 29, 32, 41, 49, 53, 54, 56, 62, 69, 72, 75, 78, 82, 85, 86, 88, 91, 93, 96, 111, 112, 115, 121, 122, 124, 125, 130, 131, 134, 135, 144, 147, 149, 151, 152, 156, 162, 164, 165, 167, 168, 171, 172, 173, 178, 180, 181, 185, 189, 192, 199, 201, 202
RSV-B	3, 4, 11, 14, 15, 18, 19, 22, 30, 32, 33, 35, 41, 48, 56, 57, 63, 67, 68, 69, 78, 82, 86, 108, 124, 126, 144, 146, 148, 149, 158, 165, 167, 172, 174, 181, 183, 184, 185, 189, 193, 194, 197, 202, 206, 208, 212, 217, 218, 220, 223, 226, 232, 235, 238, 241	1, 2, 9, 26, 34, 39, 45, 55, 60, 70, 76, 85, 87, 104, 115, 116, 122, 129, 134, 135, 137, 138, 145, 157, 159, 164, 168, 169, 171, 175, 178, 180, 182, 187, 188, 190, 192, 201, 210, 225, 227, 228, 237, 239, 240, 242

RSV diversity and substitution rates were highest in children aged 1–2 years (44.09%). These patterns were predominantly observed during winter, aligning with known seasonal trends. Severe symptoms such as shortness of breath (odds ratio: 9.229) and complications like wheezing (*P* < 0.001) were associated with higher substitution rates. Longer hospital stays were also linked (8.1 ± 4.3 vs. 6.2 ± 3.1 d; *P* = 0.034).

## Discussion

In this study, the hematological parameters showed lower white blood cell counts, neutrophil percentages, lymphocyte percentages, hematocrit, and hemoglobin levels, along with higher monocyte percentages compared to other acute illnesses. Although SAA, CRP, procalcitonin, and ketones were elevated in children with CAP, no significant differences were observed compared to other acute illnesses. These changes reflected the systemic inflammatory response triggered by viral infections, consistent with previous research.^15^

The COVID-19 pandemic significantly influenced the epidemiology of respiratory viruses, including RSV. In this study, viral detection rates reached 50.86%, peaking in 2019 and declining in 2020. This trend was likely due to public health interventions such as mask-wearing, social distancing, and school closures. These results align with earlier studies that reported a global decrease in RSV cases during the initial COVID-19 phase, followed by a resurgence as restrictions were lifted.

Consistent with previous reports,^1,2,4–7^ RSV was the most prevalent virus (26.88%), followed by IFA, PIV1, PIV3, and others. RSV was most frequently detected in toddlers aged 1–2 years. The bacterial and fungal detection rate was 7.51%, with *Haemophilus influenza* identified as the most common bacterial pathogen. It is important to note that Guangdong Provincial Hospital of Traditional Chinese Medicine was not designated for the treatment of patients with novel coronavirus infection. Therefore, this study did not include patients infected with COVID-19, and no related data were available.

Viral infections were most common during winter (16.35%). RSV activity peaked in spring, summer, and autumn, with some detections in winter. In contrast, IFA showed peak activity in winter. RSV epidemics varied annually and were disrupted by the COVID-19 pandemic in 2020. The seasonal variation of RSV observed in this study was consistent with earlier findings that RSV generally circulates during colder months but can remain active in other seasons depending on environmental and epidemiological conditions. The winter peaks observed for RSV aligned with previous studies reporting increased RSV activity during lower temperatures and higher relative humidity.^16,17^

A deeper exploration of RSV bioinformatics revealed that the evolutionary rate of the G protein-coding gene for RSV subtype A was ~2.6641 × 10^−3^ nucleotide substitutions/site/year, with a common ancestor traceable to 2002. This indicated that the ON1 genotype for subtype A in the Guangzhou region emerged as early as 2002, preceding its initial detection in China.^18^ For RSV subtype B, the evolutionary rate for the G protein-coding gene was ~2.8067 × 10^−3^ nucleotide substitutions/site/year, with a common ancestor traced to 2010.^19^ Notably, the evolutionary rate of the subtype A G protein was slightly higher than that of subtype B. Yu et al. reported substantial genetic diversity and molecular evolution in both RSV-A and RSV-B, emphasizing the G protein’s role in viral adaptation and immune evasion.^20^

Eshaghi et al. analyzed the global molecular epidemiology of RSV and identified similar evolutionary rates for the G protein gene, supporting the rapid genetic diversification of RSV-A and RSV-B.^21^ Chen et al. further demonstrated the dynamic evolution of RSV and highlighted the importance of continuous genomic surveillance to guide vaccine development and public health planning.^22^ Analysis of selective pressure sites revealed that both subtypes underwent positive and negative selection, which may influence viral pathogenesis and immune evasion mechanisms.^23^ Genetic distances at the nucleotide and amino acid levels were calculated for full-length sequences and the second hyper-variable regions of the G gene. Subtype A showed higher variability than Subtype B. Further differences were identified in the transition and transversion rates between RSV G protein genes of the two subtypes. Subtype A exhibited higher transversion rates, while subtype B had higher transition rates. These genetic variations were associated with severe clinical outcomes, including prolonged hospital stays, wheezing, and secondary bacterial infections. Similar findings have been reported, demonstrating the influence of RSV genetic variability on disease severity and pathogenesis.^20,24^

The observed genetic differences between subtypes suggest that ongoing molecular surveillance is essential for monitoring emerging strains and predicting clinical outcomes.^25,26^ The genetic diversity of RSV, particularly in ON1 and BA9 genotypes, has been widely documented and associated with the virus’s capacity to evade host immune responses.^26,27^

Progress in molecular characterization and surveillance is crucial for vaccine development and public health guidance.^20,24^ Accurate viral detection methods are necessary for reliable diagnosis and response planning. In this study, most children with pneumonia were empirically treated with antibiotics without timely adjustment. This may have resulted from limitations in viral detection techniques and the relatively short hospital stay duration (average: 6.43d). Common inflammatory markers such as CRP, PCT, SAA, and routine blood indicators did not provide effective diagnostic information for viral infections. Therefore, accurate and timely viral detection methods remain essential for clinical management.

### Strengths and limitations

This study provided a comprehensive molecular and phylogenetic analysis of RSV, detailing the genetic diversity, nucleotide substitution rates, and evolutionary patterns of the ON1 (RSV-A) and BA9 (RSV-B) genotypes. By correlating genetic variability with clinical outcomes, this study offered new insights into how RSV evolution may influence disease severity, hospital stay duration, and associated complications. In addition, the study evaluated seasonal trends before and during the COVID-19 pandemic, emphasizing the impact of public health measures on RSV circulation. These findings contribute to epidemiological surveillance, public health planning, and the development of targeted strategies, including vaccine design, to reduce RSV-associated morbidity among children.

However, the absence of comprehensive epidemiological data on pneumonia-causing viruses in the Guangzhou region limited the ability to deliver timely and effective pathogen-monitoring reports to clinicians. The continued reliance on antibiotics may reflect the unavailability of effective and reliable antiviral treatments. Another limitation was the small number of controls, as only 104 cases met the criteria for inclusion in the control group.

## Conclusion

This study provided robust regional epidemiological data on viral infections, offering clinicians valuable monitoring information for viral testing in children with CAP. The analysis focused on the relationship between RSV nucleotide variations and respiratory infection severity in children.

Our findings emphasize the higher evolutionary rate of RSV subtype B and its association with severe clinical outcomes, including prolonged hospital stays, wheezing, and secondary bacterial infections. The distinct seasonal and genetic patterns observed in this cohort underscore the need for region-specific genomic data to guide targeted interventions, including vaccine development tailored to local RSV dynamics. These results support evidence-based clinical practices, challenge conventional treatment approaches, and promote the rational use of medications to improve CAP diagnosis and care quality.

## Data Availability

The data supporting this study are available upon reasonable request but cannot be publicly shared due to privacy or ethical restrictions. Please contact the corresponding author for access.
